# Decellularized equine carotid artery layers as matrix for regenerated neurites of spiral ganglion neurons

**DOI:** 10.1177/0391398819868481

**Published:** 2019-08-22

**Authors:** Suheda Yilmaz-Bayraktar, Jana Schwieger, Verena Scheper, Thomas Lenarz, Ulrike Böer, Michaela Kreienmeyer, Mariela Torrente, Theodor Doll

**Affiliations:** 1 Department of Otolaryngology, Hannover Medical School, Hannover, Germany; 2 Lower Saxony Centre for Biomedical Engineering, Implant Research and Development (NIFE), Hannover, Germany; 3 Cluster of Excellence Hearing4All, Hannover, Germany; 4 Department of Cardiothoracic, Transplantation and Vascular Surgery, Hannover Medical School, Hannover, Germany; 5 Department of Otolaryngology, Faculty of Medicine, University of Chile, Santiago, Chile; 6 Fraunhofer Institute for Toxicology and Experimental Medicine, Hannover, Germany

**Keywords:** Nerve–electrode interface, cochlear implants, guiding structures, biomaterials, decellularized matrix

## Abstract

Today’s best solution in compensating for sensorineural hearing loss is the cochlear implant, which electrically stimulates the spiral ganglion neurons in the inner ear. An optimum hearing impression is not ensured due to, among other reasons, a remaining anatomical gap between the spiral ganglion neurons and the implant electrodes. The gap could be bridged via pharmacologically triggered neurite growth toward the electrodes if biomaterials for neurite guidance could be provided. For this, we investigated the suitability of decellularized tissue. We compared three different layers (tunica adventitia, tunica media, and tunica intima) of decellularized equine carotid arteries in a preliminary approach. Rat spiral ganglia explants were cultured on decellularized equine carotid artery layers and neurite sprouting was assessed quantitatively. Generally, neurite outgrowth was possible and it was most prominent on the intima (in average 83 neurites per spiral ganglia explants, followed by the adventitia (62 neurites) and the lowest growth on the media (20 neurites). Thus, decellularized equine carotid arteries showed promising effects on neurite regeneration and can be developed further as efficient biomaterials for neural implants in hearing research.

## Introduction

The inner ear consists of the vestibular organ and the cochlea, which grows helically around its axis, the modiolus, and comprises blood vessels and the bipolar spiral ganglion, which forms the auditory nerve. The cochlea is composed of three fluid-filled compartments, the scala tympani, scala media and scala vestibuli, and the organ of Corti.^1^ The cell bodies of spiral ganglion neurons (SGN) are located in the Rosenthal’s canal in the modiolus. Their dendrites extend from their cell bodies through the modiolus and the osseus spiral lamina and form the synaptic contacts with hair cells in the organ of Corti, which is located in the scala tympani. The death or degeneration of hair cells can lead to deafness, because the downstream SGN are no longer stimulated.^2^ The function of the lost sensory hair cells can be replaced by a cochlear implant (CI). CIs can support deaf patients, whose auditory nerves are still intact. It consists of an implantable electrode carrier and a processor. After inserting the CI electrode into the scala tympani of the cochlea, neurons can be stimulated electrically to create a hearing impression,^3^ which, however, is not comparable to the physiological hearing impression. CIs are still facing challenges, such as the suboptimal nerve–electrode interface.^4^ The longitudinal propagation of electric fields causes a suboptimal electrical channel separation because the anatomical gap between the perilymph-surrounded electrode contacts in the scala tympani and the neurons located in the Rosenthal’s channel of the bony modiolus is bridged insufficiently.^5–7^ Yet, through self-bending electrode shafts, the positioning of electrodes closer to the modiolus is possible. This reduces the distance between electrodes and neuronal tissue and therefore reduces the stimulation threshold, but is not a significant improvement for channel separation. This is due to long current propagation in the perilymph, through which neurites cannot grow.^8^ For a direct contact between neurons and electrodes, neurites need to grow and sprout out from the bony structures into the perilymph-filled gap and have to be guided to the electrode surface. In order to support the regeneration and guiding of neurites, possible guiding materials with different surface structures are under research, due to their influence on neurite outgrowth behavior and subsequently may improve the CI performance.^9–11^ In particular, bioartificial materials like cellfree extracellular matrices might be promising materials to support neurite outgrowth in CI. Decellularized equine carotid arteries (dEAC) might be a promising candidate for this. A detailed protocol and characterization of the here-tested dEAC has been described previously by Böer et al. The scaffolds were decellularized by a detergent-based approach and thoroughly characterized with respect to their residual proteins,^12,13^ their biocompatibility in vitro^14,15^ and in vivo^16^ and their biomechanical properties.^14^ It was shown that for complete removal of cellular components and a maximized biocompatibility in vitro and in vivo an intensified decellularization process was necessary.^13,14,16^ Yet, biomechanical properties were maintained and the scaffold structure was preserved.^13,14^ This structure comprises three distinct layers of equine carotid arteries (EAC): the outer tunica adventitia is known to display a randomized pattern, while the tunica media displays a structure based on parallel elastin fibers and the tunica intima has almost no pattern.^17,18^ Moreover, intensively (d)EAC have been shown to be recellularized, with human cells,^14^ and remodeled in vivo to an extremely high extent.^16^ By these investigations and since the structure of theses layers differ considerably, dEAC most likely are highly promising for cell adhesion and support of neurite growth. Thus, we here tested dEAC as matrix for SGN and analyzed the outgrowth behavior of SGN-neurites. The three layers of dEAC were tested separately, with respect to adhesion (in terms of cell growth on surface) and neurite outgrowth, to get insight into favorable structures of dEAC for auditory neurons and therefore the suitability as alternative biomaterial for the application in hearing research to improve the CI treatment.

## Materials and methods

### dEAC

**Figure 1.**
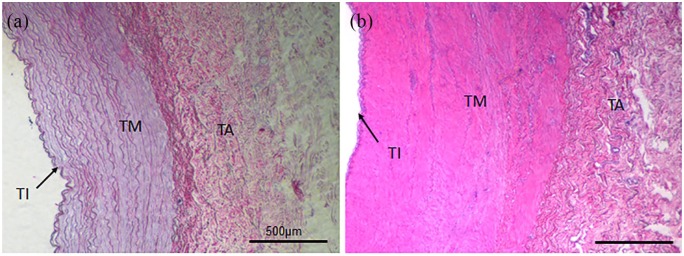
Histology of intensified decellularized equine carotid arteries (EAC). EAC were decellularized by an intensified detergent-based protocol described by Böer et al. and paraffin sections were stained by hematoxylin and eosin staining (a). The three-layered structure of native arteries (b) composed of tunica intima (TI), tunica media (TM), and tunica adventitia (TA) is indicated.

EAC were obtained from a local slaughterhouse under semi-sterile conditions and stored in cooled 0.9% sodium chloride (NaCl) + 1% penicillin/streptomycin until further processing. The adjacent tissue was carefully removed. EAC pieces of 10 cm were thread onto rings of Teflon tubes to prevent collapsing of the arteries and improve purging. Rings with the carotids were disinfected with 70% ethanol for 20 min and washed with 0.9% NaCl. For decellularization, EAC were transferred into 300 mL decellularization solution containing 0.5% sodium dodecyl sulfate and 0.5% sodium deoxycholate and shaken for 72 h. After intense washing with distilled water and 0.9% NaCl, EAC were treated with 75 U/mL endonuclease (Merck, Darmstadt, Germany) for 4 h at 37°C. Furthermore, EAC were washed with 0.9% NaCl and stored at 4°C.^12–14^ Subsequently, dEAC pieces were cut into three different layers using fine scissors and 3 mm × 3 mm pieces were placed in 96-well plates. In Figure 1, the histology of intensified dEAC and its three-layered structures are shown before and after the decellularization process.

### Preparation of spiral ganglion explants

Cell experiments were performed with spiral ganglia explants (SGE) in accordance with Schwieger et al.^19^ Sprague Dawley rats (2–4 days old) were decapitated, skulls were opened midsagitally, and the temporal bones were collected. The cochleae were exposed and the membranous labyrinth was dissected by peeling off the bony cochlear capsule under a dissection microscope. After removing the stria vascularis and the organ of Corti, the spiral ganglion was isolated. Finally, the spiral ganglion was divided into three smaller SGE that were placed on the dEAC layers. On each layer, one major and one smaller SGE were placed. Besides the SGN, the neuron-associated glia cells and fibroblasts of the Rosenthal’s canal were also part of the explanted cell cluster.^19–21^

### Cultivation of the SGE

One or two SGE were placed onto the abluminal side of the adventitia and the luminal side of the intima and media. The three different dEAC layers with SGE were cultivated for 5 days in a medium consisting of Panserin 401 (PAN-Biotech, Aidenbach, Germany) supplemented with insulin (8.7 µg/mL; Biochrom, Berlin, Germany), penicillin (30 U/mL; Biochrom), glucose (0.15%; B. Braun Melsungen, Melsungen, Germany), phosphate-buffered saline (PBS; Ca^2+^/Mg^2+^-free PBS; 0.172 mg/mL; Gibco by Life Technologies, Carlsbad, CA, USA), HEPES buffer solution (23.43 µM; Invitrogen, Carlsbad, CA, USA) and N-2 supplement (0.1 µL/mL; Invitrogen) in an incubator (CB 150 E3; Binder, Tuttlingen, Germany) at 37°C, 5% CO_2_ and humidity of 95%. The serum-free medium was conditioned with 10% fetal calf serum (FCS). At the end of the experiments, dEAC layers with SGE were fixed with a 4% paraformaldehyde (PFA) solution per well for 1 h at 4°C and washed two times using hydroxymethyl-aminomethane (TRIS) buffer (Merck) and stored at 4°C.^22^ The experiments were repeated in triplicate with SGE from different animals for each of the layers in one-well plate.

### Immunocytochemistry

**Figure 2.**
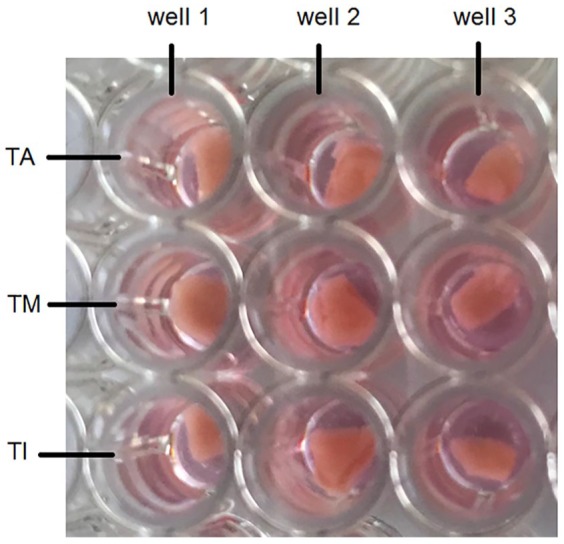
SGE placed onto the three dEAC layers: tunica adventitia (TA), tunica media (TM), and tunica intima (TI), which were placed in a 96-well plate. Per well, 200 μL of culture medium were added. Three repetitions per layer were performed in the experiment.

The neurite outgrowth was assessed manually for the SGE on each layer (Figure 2). Sprouted neurites on the tunica surfaces and neurites which grew into the dEAC layers were counted. A neuron-specific staining was performed to evaluate neurite outgrowth by recording confocal laser scanning microscope (CLSM) images with the Leica TCS SP8 stimulated emission depletion (STED) microscope using the confocal imaging mode (objectives: HC PL FLUOTAR 5×/0.15 DRY and HC PL FLUOTAR 10×/0.30 DRY). The neurofilament-specific staining was adapted from Schwieger et al.^19,22^ The dEAC layers and the SGE were permeabilized with a permeabilization solution (Sigma–Aldrich, St. Louis, MO, USA) containing 0.5% Triton X (Sigma–Aldrich) and TRIS and then blocked using a blocking solution containing 10% serum (FCS; Biochrom) and bovine serum albumin (BSA; Sigma–Aldrich) and 1% Triton. To distinguish SGN from non-neuronal cells, they were stained using a polyclonal antibody against the 200 kD neurofilament (1:1000; Abcam, Cambridge, UK) in combination with the secondary antibody Dylight 488 goat-versus-chicken (Abcam). 4′,6-Diamidino-2-phenylindole (DAPI; Abcam) was used for nucleus labeling.

### Microscopic and statistical analysis

**Figure 3.**
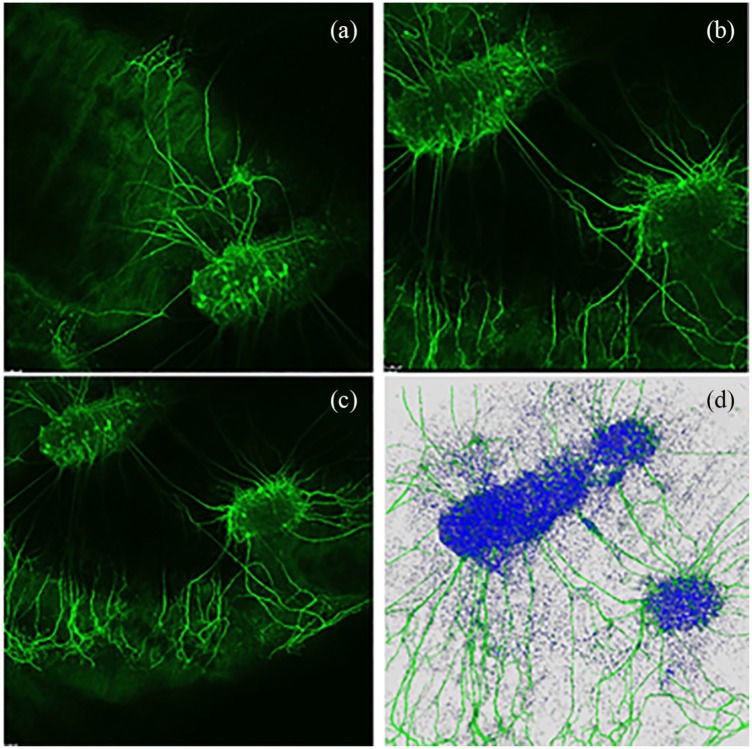
Exemplary z-stack and superimposed 3D image of neurite growth on dEAC layer. (a)–(c) Several images of an area. (d) Interference of the several images to a superimposed 3D image.

After the immunocytochemistry, the tissue layers were placed on coverslips so that CLSM images could be recorded distinctly and microscopic analyzes performed. Each tissue layer with all three replications was analyzed separately. First of all, the explants on the tissue were identified to get an overview of the observed area. Mostly, one bigger explant and an additional smaller explant were cultured on each piece of tissue. Usually the neurites grew into different penetration depths of the 3 mm × 3 mm pieces of the dEAC layers. Due to the three-dimensional (3D) growth of the neurites, z-stacking was performed to achieve 3D images. For that, one beginning edge of the explant was selected and recorded. The surrounding area was recorded as well because usually, neurites sprouted out of the explants in to the decellularized tissues. Thus, typically, the selected edge of the last z-stack was up to the neurite growth trend. The z-stack setting of the microscope recorded several images (Figure 3(a)–(c)) of this area and interfere them to a superimposed image (Figure 3(d)). Several replicates and fields of view were recorded per dEAC layer. In this way, the complete growth behavior of the neurites could be followed. After taking the CLSM images, they were evaluated for the number of neurites growing into the respective layers. The areas of the explants were measured manually, which is a well-established method to analyze microscopic images by counting the total number of cells or neurites and measuring the neurite length in millimeters with a ruler.^11,23^

Furthermore, it has to be mentioned that, this study focuses on feasibility analysis of dEAC as growth matrix for regenerated neurites of auditory neurons. Therefore, the study includes only three repetitions. The averages of the three replicates were tested by Kruskal–Wallis test. Significances for non-parametrical values and a normal distribution were not found. In order to normalize the counted number of neurites to the SGE sizes and to analyze a possible dependency, the areas of the placed explants were calculated. Spearman’s correlation was determined, while significances were not found. All analyses were performed with GraphPad Prism^®^ 5 software.

## Results

As stated in the “Introduction” section, the spiral ganglion cell culture is well-established for analyzing the neurite growth behavior. However, biocompatibility tests on the dEAC were performed previously by Böer et al. In this approach, both already established systems were combined and the neurite regeneration was analyzed. Our results are closely aligned to Tuft et al. which will be discussed further in the discussion part.

**Figure 4.**
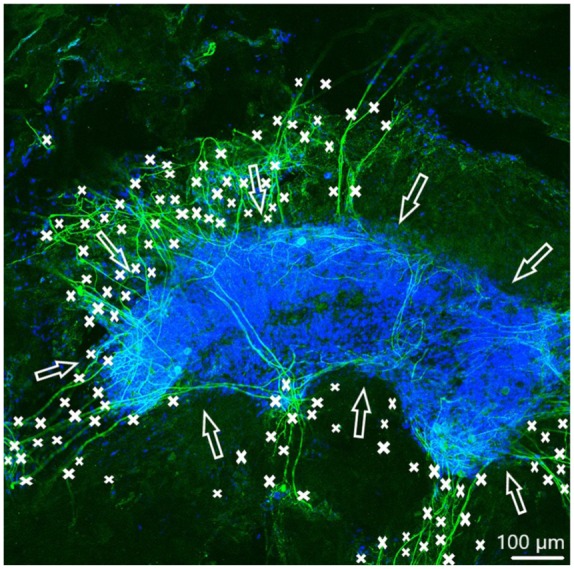
Spiral ganglion explants cultivated on the tunica adventitia (not visible). CLSM image shows neurite outgrowth (neurofilament, green). DAPI (blue) illustrates the nuclei of all cells of the SGE. Neurites are marked by asterisks and arrows pointing on the surface of SGE. Approximately 119 neurites sprouted onto the tunica were counted.

**Figure 5.**
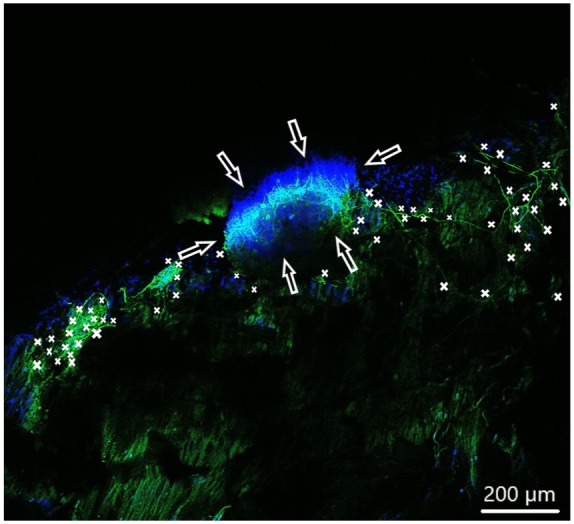
Spiral ganglion explants cultivated on the tunica media (not visible). CLSM image shows neurite outgrowth (neurofilament, green). DAPI (blue) illustrates the nuclei of all cells of the SGE. Neurites are marked by asterisks and arrows pointing on the surface of SGE. Shown are fewer neurites about 60 sprouted into the matrix.

**Figure 6.**
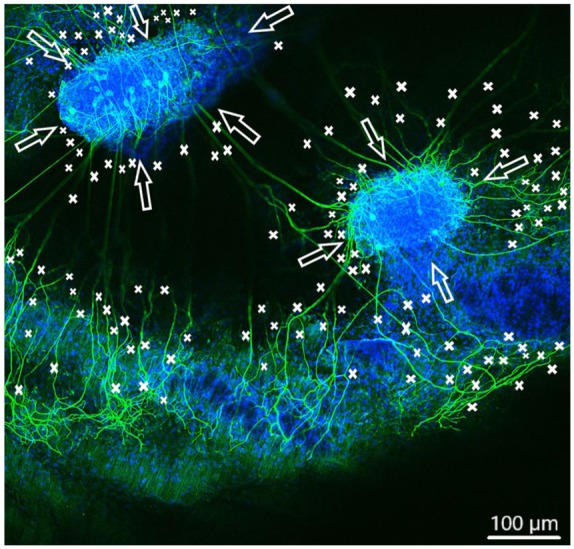
Spiral ganglion explants cultivated on the tunica intima (not visible). CLSM image shows neurite outgrowth (neurofilament, green). DAPI (blue) illustrates the nuclei of all cells of the SGE. Neurites are marked by asterisks and arrows pointing on the surface of SGE. Totally over 150 neurites sprouted out of both SGE in almost every direction.

**Figure 7.**
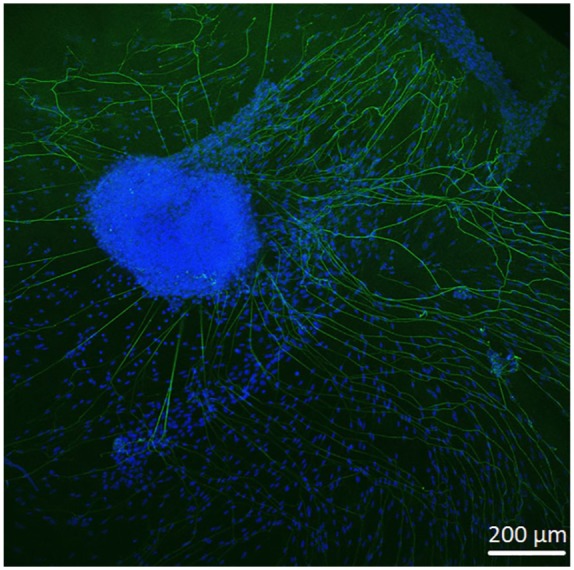
Control image showing two-dimensional neurite growth of SGE on a glass slide.

In order to observe growth characteristics of SGE neurites on the tunica adventitia, intima, and media, we focused on sprouted neurites into the tunica matrices. Cell adhesion in terms of SGE attachment to the relevant tunica and generally, neurite outgrowth of cultivated SGE were observed in the surface of all three dEAC layers (Figures 4–6). Nuclei of the cells were labeled by DAPI (Figures 4–6, blue) and allowed the assessment of the location of adhered SGE, indicated by arrows. Partly, neurites grew into an adjacent SGE and regenerated neurites extended to the edges of the tunica (Figure 6). In some areas, the neurite sprouting could not be clearly followed due to deeply invading the matrix of neurites (Figure 5–6). We determined which of the dEAC layers were most supportive for neurite regeneration and sprouting by counting the number of sprouted neurites per SGE. Apart from the number of neurites, we calculated the SGE area by measuring the lengths and the widths. The SGE heights are anatomically similar, while the SGE sizes are determined by its area and not volume. Three wells, each with one piece of the tunica adventitia, media, and intima, and SGE were cultivated in one-well plate and finally nine samples were analyzed in total. In the first well, we observed a major SGE and a smaller one on the tunica adventitia (Table 1). Some neurites sprouted out of the main SGE and grew in the direction of the smaller SGE. Approximately 64 sprouted neurites were counted in the first well. Figure 4 shows an exemplary image of SGE on the tunica adventitia of the second well where over 119 regenerated neurites were counted in the SGE, while four sprouted neurites were counted in the third well on the SGE. Figure 5 shows a SGE on the tunica media. About 60 sprouted neurites into the matrix were counted while the majority of neurites on this tunica within the SGE did not grow out and was therefore not counted. However, these neurites were notable long about up to 600 µm. The SGE on the tunica media in well 2 was likewise low and we counted about 20 neurites. It should be noted that, in well 3, the SGE on this tunica could not be found, while the number of neurites could not be counted and is thus 0. CLSM images of SGE incubated on the tunica intima showed a higher number of sprouted neurites compared to the tunica adventitia and tunica media. Figure 6 illustrates the neurite growth behavior on the tunica intima in the second well. Again, neurites grew between the two examined adhered SGE. In the third well on the tunica intima about 100 neurites were counted on the SGE. Similar to the tunica media, in the first well on the intima, the SGE could not be found after the staining procedure, while the number of neurites could not be counted and thus is set to 0. Figure 7 demonstrates the control of the results shown in Figures 4–6. Since no standard is defined for a positive or negative control for 3D matrix, the control image shows the two-dimensional (2D) neurite growth on a glass slide.

**Table 1.** Overview of the explant areas and number of neurites of each tunica on each well.

	Explant area (µm^2^)	No. of neurites
Adventitia well 1	60,000	64
Adventitia well 2	60,000	119
Adventitia well 3	20,000	4
Media well 1	60,000	60
Media well 2	80,000	20
Media well 3	0	0
Intima well 1	0	0
Intima well 2	80,000	150
Intima well 3	90,000	100

### General evaluation and correlation analysis

**Figure 8.**
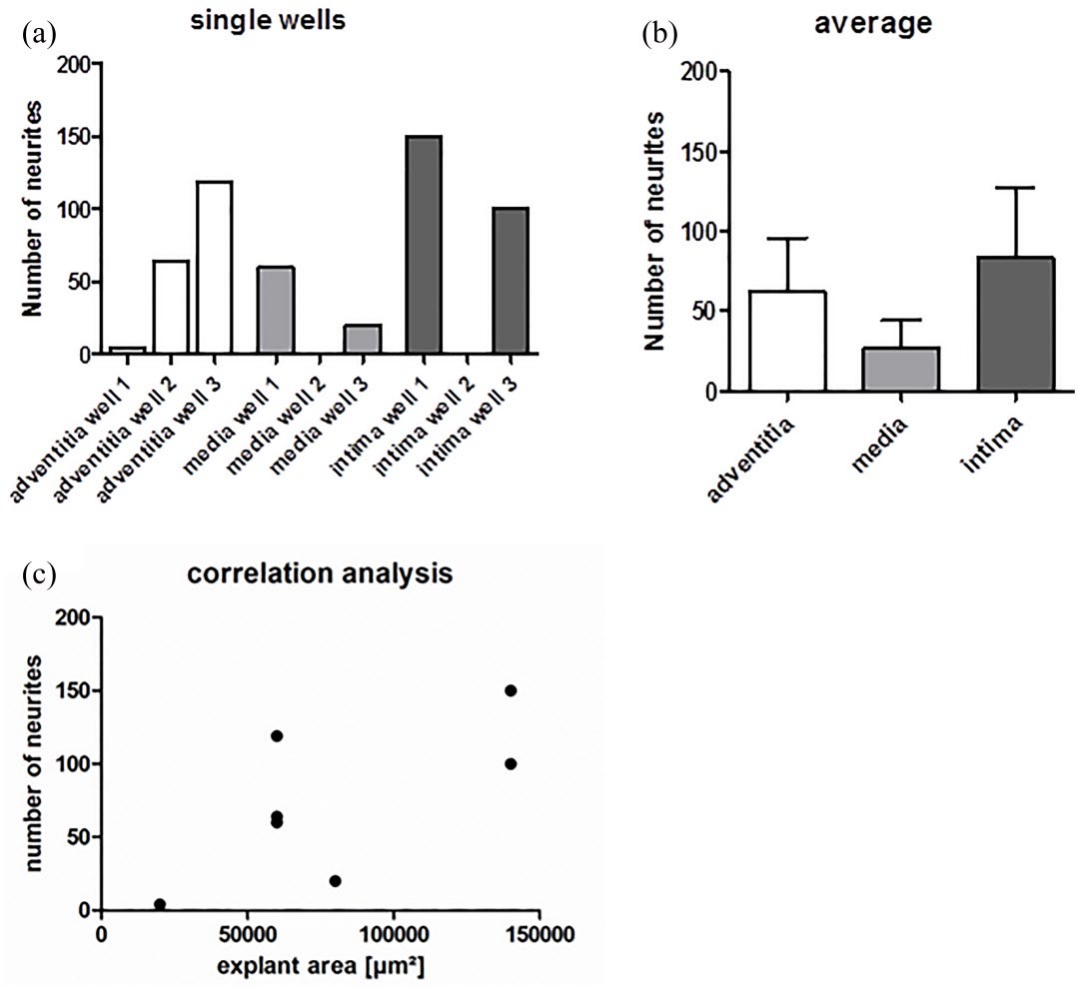
(a) Number of sprouted neurites into the tunica adventitia, media, and intima in single wells. (b) The averages of the three replicates. Shown are means ± SEM, significances tested by Kruskal–Wallis test for non-parametrical values were not found. All analyses were performed with GraphPad Prism^®^ 5 software. (c) To analyze a possible dependency between detected number of neurites and the explant area which were placed onto the tunicas, Spearman’s correlation was performed with GraphPad Prism 5 software. A *p*-value more than 0.05 was considered as there are no significant differences between the tested conditions. Spearman’s correlation is 0.468 which was analyzed as a low positive correlation between SGE size and the number of neuritis.^32^

Figure 8(a) gives an overview on the number of outgrowing neurites in single wells on each of the three dEAC layers, while in Figure 8(b), the number of neurites on the three dEAC layers is shown in average. Between the tunica adventitia and tunica intima, only slight differences were appreciable. The maximum value of regenerated neurites was determined on the tunica adventitia over 119 and on the tunica intima over 150 neurites. In comparison with the tunica adventitia and the tunica intima, neurite growth on the tunica media was diminished, which clearly showed the lowest maximum value of sprouting with approximately 60 sprouted neurites. Focusing on the averaged results of the experiments, we could determine that the highest number of neurites extended on the tunica intima (83 neurites), followed by the tunica adventitia (62 neurites). The lowest average number of regenerated neurites was observed on the tunica media (27 neurites) (Figure 8(b)). Significances tested by the Kruskal–Wallis test for non-parametrical values were not found. In Table 1, the explant areas and number of neurites of each tunica in each well are presented as an overview. According to the correlation analysis (Figure 8(c)), there is only a low positive correlation between the number of sprouted neurites and the area of the placed SGE, as Spearman’s correlation of 0.468 was considered. However, significances in correlation were not found.

## Discussions

In order to contribute an approach for the nerve–electrode interface, the CI research is concerned with questions regarding biological and well-biocompatible materials, which support the regeneration of neurites. On the basis of the facts explained in the “Introduction” section, we believe that the different structures and morphology of the vessel layers, especially the smooth wall toward the blood flow and the reticular tissue inside, might be differently suitable for cell adhesion and neurite outgrowth. This is why this proof-of-principle study was intended to evaluate the suitability of the three layers tunica adventitia, media, and intima of dEAC separately as matrix for neurons, focusing on neurite outgrowth. In our investigated results, we could show for the first time neuron adhesion and neurite sprouting on the dEAC and confirm this thesis. In the following, the rationale for choosing this specific experimental setup with three layers and the possible relationship with neurite behavior will be discussed in detail.

### dEAC

In current implant research, extracellular matrices are used as biological biomaterial scaffold due to their high biocompatibility. Next to this, properties that make it more appropriate for clinical application are viscoelasticity and the ability to support cell attachment through collagen, fibronectin, and laminin.^24^ dEAC have been thoroughly characterized in the past considering biomechanical properties and cytotoxicity,^14^ in vivo biocompatibility^16^ and functionality as vascular graft.^25^ Moreover, also an excellent reseeding capability of the cellfree scaffolds was evaluated^14^ most likely due to the unaffected collagen contents. The study concluded that the decellularization generates matrix scaffolds which are highly suitable for tissue engineering.^14^ Also, a highly satisfying biocompatibility of dEAC has been demonstrated by Jeinsen et al.^16^ in an in vivo evaluation using rat subcutaneous implantation of dEAC that demonstrated low inflammatory infiltration, a fast and substantial neovascularization, and neglectable fibrosis. It was shown by a proteomic approach, that the complete removal of cells including cellular components is not possible with conventional processes.^12^ Therefore, an intensified detergent-based protocol was established whereby cellular components were removed extensively and immunogenicity was reduced as well.^13^ Structural proteins like collagen or elastin may be damaged by processes with stringent treatments, which may cause a reduction of scaffold stability,^26^ which was, however, not the case with dEAC.^14^ During the decellularization process, it is required to preserve the composition of the extracellular matrix, such as its native structures.^24,27^ Another essential aspect is the biocompatibility, which can be influenced by the manufacturing protocol because of possibly affecting the cytotoxicity through residual detergents.^28^ Equally, the disinfection of decellularized tissues may result in the same effect.^15^ Finally, it has to be noted that, decellularization protocols significantly influence the scaffolds biomechanical composition, the efficiency of cell removal, tissue ultrastructure, and mechanical behavior as well.^29^ However, the intensified dEAC also seem to be a promising biomaterial for the support of neurite outgrowth as needed for bridging the gap between the electrodes and auditory neurons in CI-patients.

### Microscopic and statistical analyses

Due to the different compositions and morphologies, the three layers were investigated separately. Evaluations of CLSM images were performed to record cell adhesion and neurite growth out of the SGE onto and into the different dEAC layers. In spite of CLSM images superimposed into different penetration depths, it was difficult to count exactly the number of regenerated neurites because of overlapping growth of neurites. Moreover, it should be noted, that only vague statements can be made with respect to the number of neurites due to the major variance and few repetitions of trial, which also was confirmed by the Kruskal–Wallis test as there was no significances. Apart from that, we detected areas on the three dEAC layers where neurites sprouted massively, but also areas where no growth was detected. In order to exclude a relationship between the different number of neurites and the sizes of the placed SGE, we performed a correlation analysis by calculating Spearman’s correlation coefficient which was 0.468. This correlation coefficient is evaluated as a low correlation^30^ between the SGE size and the number of neurites. Due to the low correlation between the SGE sizes and the numbers of neurites, the varying numbers of neurites can be traced back to different structures of the three dEAC layers which will be discussed below.

### Neurite growth on dEAC

First of all, we could show that SGE adhesion and neurite growth was possible on each tissue layer. Even so, we observed a high diversity among the numbers of sprouting between the different preparations of each layer for the repetition wells. This may be due to both differences in the dissected SGE (e.g. used animal, damage of cells during preparation, basal or apical part of the spiral ganglion) and varying components and structures of the respective tunica. Tuft et al. have investigated neural pathfinding behavior of neurites on parallel line-space gratings and on multidirectional and angled patterns. They observed that on non-patterned surfaces, neurites oriented randomly and consistently imitated and tracked the characteristics of the unidirectional micropatterns.^31,32^ Compared to this, neurites did not consistently track the repeating angled pattern. Contrary to this, neurites extend to avoid turning events so that multiple feature transitions can be crossed-over. Apart from this, Tuft et al. have detected that neurite lengths are 20% shorter on multidirectional substrates compared to unidirectional patterns while neurites branching and crossings are significantly higher. This suggests that neurites generally prefer long extensions on straight and parallel structures, while they prefer branching crisscross trends on angled or crossing patterns.^31,32^ In general, our findings reflect the results of Tuft et al. As we indicated in the results of the tunica media, SGE on this matrix extend up to 600 µm long, although the number of neurites in general was decidedly low. However, neurites on the tunica adventitia and intima were shorter but they sprouted much more branching and crossing. The shorter neurite sprouting on angled and crossing patterns is traced back to the higher potential of encounters with feature edges on the neural pathway compared to fewer encounters on smooth or unidirectional surfaces.^31,32^ This could also be an explanation for the longer neurite extension on the tunica media than on the tunica adventitia and intima, since the different layers of the dEAC distinguish in their tissue structures. These differences may have influenced the neurite regeneration in our experiments and will be discussed in the following.

### The tunica adventitia, media, and intima

In order to relate the general composition and morphology of the arteries with neurite outgrowth, the presented results of each tunica will be discussed in the following. Generally, neurite growth was mostly on the tunica intima, followed by the tunica adventitia, and the lowest mean neurite sprouting was detected on the tunica media.

The tunica adventitia contains connective tissue: elastin and collagen, fibroblasts, mast cells, macrophages, and occasional Schwann cells with associated nerve axons.^33,34^ Interestingly, the nerves are only contained in the adventitia and do not penetrate the tunica media^35,36^ which is bounded on the luminal side by an internal elastic lamina. Within the tunica media, there are no elastic laminae and smooth muscle cells are connected with few collagenous fibrils between cells.^37^ The most of the elastin, which is arranged in fenestrated lamellae between which are collagen fibers, thin layers of proteoglycan-rich extracellular matrices and smooth muscle cells, make up the tunica media.^38,39^ Elastin is distensible and has a low tensile strength.^17^ The luminal surface of arteries, called the tunica intima, is lined with endothelial cells,^40^ which produce and attach to a basal lamina supported by the internal elastic lamina.

On the tunica adventitia we observed a tendency toward higher number of neurites. If we compare the mean number of neurites on the tunica adventitia with that on the tunica intima, the lower number of neurites on the tunica adventitia may be related to its disordered structures typical for vascularized connective tissue. However, this can be explained by the investigations of Tuft et al.^31,32^ that neurites on disordered structures prefer multiple crossed-over branching, which may cause a high number of neurites. On the tunica media, several cells and grown neurites within the SGE were observed. In spite of that, here, the number of neurites is lower than that on the tunica adventitia and intima. This observation could be related to a higher immunogenicity of this layer. Böer et al. demonstrated that the immunogenicity of dEAC in mice could be traced back to single molecules. The most prominent one was collagen VI which is interestingly localized in the so-called oxytalan fibers of the tunica media. Most likely, the dissections of the dEAC to isolate the tunica media led to an exposure of collagen VI to the cells of the SGE and the collagens’ immunogenicity may be responsible for the impairment of growth and sprouting.^13^ A further reason for the low number of neurites on the tunica media could be the distensible elastin with low tensile strength,^17^ which possibly inhibits the survival of neurons and thus the outgrowth of several neurites. In contrast to the more randomized structure of the tunica adventitia, the intima displays a smooth surface that basically consists of the basal membrane. This structure particularly seems to facilitate the adhesion and the outgrowth. Furthermore, the high suitability of the tunica intima for neurite outgrowth is supported by its low immunogenicity and high biocompatibility, which has been observed recently by Jeinsen et al.^16^ Moreover, the intact basal lamina of the tunica intima provides multiple cell adhesion sites for endothelial cells^41^ which most likely also convey the adherence of neuronal cells, since they use the same structures. For example, the glycoprotein laminin has to be emphasized. It belongs among others to the neural extracellular matrix. Laminin promotes the adhesion and migration of developing neural cells^42,43^ via the laminin-binding integrin receptor α6β1,^44^ whereas vascular endothelial cells using β1 and β3 integrin receptors for their adherence to laminin,^45^ which has previously been used as neuroprotective factor for nerve regeneration in rats.^46^ Also Nagy et al.^47^ have shown that endothelial cells provide a substrate for the proliferation of enteric neural cells via β1 integrins. Thus, we hypothesize that the increased neurite outgrowths on the tunica intima may be due to, on one hand, the presence of laminin in the matrix and, on the other hand, the support by endothelial β1 integrins which most likely resisted the detergent-based decellularization process.^12^ At least, it should be mentioned that both—on the tunica adventitia and intima—neurites sprouted in the direction of other SGE (Figure 5 and 6) which may be due to the stimulating effect of the included non-neuronal cells which most likely release growth factors.^47^

### Outlook

There are currently few other approaches using decellularized matrices to support neuronal outgrowth. Nerve constructs consisting of decellularized vein grafts filled with spider silk fibers as a guiding material were used to bridge nerve defects. In this approach, in the first place, spider silk was shown to be a viable guiding material for migration and proliferation of Schwann cells and axonal re-growth as well, and also the extracellular matrix was highly suitable to support peripheral nerve regeneration.^48^ Inspired by this approach, one possibility would be the insertion of dEAC layers in CIs as gap junctions since they have excellent stability. Huang et al. showed that conduits, consisting of a hollow highly porous cylindrical sheath composed of regenerated mulberry silk fibroin and containing longitudinally oriented luminal Spidrex fibers coated with hyaluronic acid, act as guiding materials to bridge the gaps of defect nerves as well. According to their results, the conduits containing luminal fibers of the so-called “Spidrex” were supporting neurite outgrowth.^49^ Thus, extracellular matrices have been shown to support neuronal growth. Our study adds the information that most probably the tunica intima and the tunica adventitia are layers responsible for this effect. Among other approaches, the both layers could be stripped from the dEAC, homogenized and added to the CI as an adhesion factor in the form of denominational tissue (to benefit from the structures of the dEAC) or powder (use of adhesion molecules of dEAC) to stimulate the SGN in the direction of the electrodes. A possible negative effect of the dEAC, which has to be kept in mind, is the risk of an enhanced encapsulation of electrodes with connective tissue due to the dEAC. This could isolate the stimulating CI electrode and increase the current spread. It is possible to object to this that the outgrowth of the neurites was mostly longer than the DAPI-positive non-neuronal cells, indicating a direct, not glia cell-mediated growth of the neurites on the dEAC. Apart from that, we are interested in the elicitors of the regeneration process to probably integrate this in the CI system. Certainly, an insertion of dEAC structures in CIs has to be combined with other growth matrices, through which only the neurite growth is induced. However, for an improved CI treatment supporting structures are needed in the perilymph-filled scala tympani. Moreover, it has been shown that in case of neurite growth in connective tissue to the implant, the tissue does not necessarily have a negative impact on the neurite growth because the SGN are stimulated directly via their neurites.^50^

## Conclusion

The explant adhesion and neurite sprouting were supported by the dEAC layers tested as growth matrix for SGE. Especially, the tunica intima and adventitia showed promising effects on the neuron outgrowth. The presented results are one first step to a gapless nerve–electrode interface and at the moment far away from a clinical translation. Nevertheless, we could show, for the first time, that the neurite regeneration of auditory neurons can be supported by dEAC and that there were differences between the different layers.

This approach is important not only for the hearing research but also for other neural prostheses. Further investigations, focusing on the differences of the dEAC layers regarding their influence on and interaction with neurons, might lead to a future solution to bridge the anatomical gap between neurons and implant.
